# Oxygen dynamics in shelf seas sediments incorporating seasonal variability

**DOI:** 10.1007/s10533-017-0326-9

**Published:** 2017-03-29

**Authors:** N. Hicks, G. R. Ubbara, B. Silburn, H. E. K. Smith, S. Kröger, E. R. Parker, D. Sivyer, V. Kitidis, A. Hatton, D. J. Mayor, H. Stahl

**Affiliations:** 1 Scottish Association for Marine Science, Scottish Marine Institute, Oban, Argyll, PA37 1QA UK; 2 Centre for Environment, Fisheries and Aquaculture Science, Lowestoft, NR33 0HT UK; 3 Ocean Biogeochemistry and Ecosystems, National Oceanography Centre, Southampton, SO14 3ZH UK; 4 Plymouth Marine Laboratory, Prospect Place, The Hoe, Plymouth, PL1 3DH UK; 5 College of Sustainability Sciences and Humanities, Zayed University, Dubai, United Arab Emirates; 6 Present Address: Department of Chemistry, University of Glasgow, University Avenue, Joseph Black Building, Glasgow, G12 8QQ UK

**Keywords:** Oxygen consumption, Benthic carbon cycling, Total oxygen uptake, Shelf sea, Benthic biogeochemistry, Benthic mineralisation

## Abstract

Shelf sediments play a vital role in global biogeochemical cycling and are particularly important areas of oxygen consumption and carbon mineralisation. Total benthic oxygen uptake, the sum of diffusive and faunal mediated uptake, is a robust proxy to quantify carbon mineralisation. However, oxygen uptake rates are dynamic, due to the diagenetic processes within the sediment, and can be spatially and temporally variable. Four benthic sites in the Celtic Sea, encompassing gradients of cohesive to permeable sediments, were sampled over four cruises to capture seasonal and spatial changes in oxygen dynamics. Total oxygen uptake (TOU) rates were measured through a suite of incubation experiments and oxygen microelectrode profiles were taken across all four benthic sites to provide the oxygen penetration depth and diffusive oxygen uptake (DOU) rates. The difference between TOU and DOU allowed for quantification of the fauna mediated oxygen uptake and diffusive uptake. High resolution measurements showed clear seasonal and spatial trends, with higher oxygen uptake rates measured in cohesive sediments compared to the permeable sediment. The significant differences in oxygen dynamics between the sediment types were consistent between seasons, with increasing oxygen consumption during and after the phytoplankton bloom. Carbon mineralisation in shelf sediments is strongly influenced by sediment type and seasonality.

**Electronic supplementary material:**

The online version of this article (doi:10.1007/s10533-017-0326-9) contains supplementary material, which is available to authorized users.

## Introduction

Continental shelf sediments play a vital role in biogeochemical cycling, accumulating and burying organic matter (Jahnke et al. 2005; Woulds et al. 2007). Shelf sediments receive up to 50% of primary productivity from the overlying surface waters (Stahl et al. 2004a), and as this source of carbon reaches the benthos it is recycled, mineralised or buried (Canfield et al. 1993; Glud 2008). One of the determinants of burial efficiency of organic matter is the presence and the depth of oxygen penetration (Burdige 2007; Glud 2008; Woulds et al. 2007). The ability to sequester organic carbon is influenced by the presence and activity of macrofauna and meiofauna (Woulds et al. 2007), and the type of sediment (cohesive vs permeable (Fuchsman et al. 2015; Glud 2008)). The efficiency of organic carbon burial has been directly linked to the pore water oxygen concentration and exposure time (Hartnett et al. 1998), and the availability of oxygen regulates different benthic biogeochemical processes (Glud 2008; Klar et al. 2017; Scholz et al. 2014; Serpetti et al. 2016). Oxygen distribution in sediments influences denitrification, trace metal speciation and release of iron in pore waters (Kitidis et al. 2017; Klar et al. 2017; Rabouille et al. 2003; Thompson et al. submitted, this issue). In coastal sediments and shallow waters, oxygen production through photosynthesis may exceed consumption. However, in sediments below the photic zone, oxygen consumption exceeds production, due to the absence of photosynthesis, and in this case, the distribution and consumption of oxygen will vary with sediment type and the supply of organic matter from the surface waters (Stahl et al. 2004a). The degradation and production of organic matter in the sediment can often be many orders of magnitude higher than the surface or overlying waters (Glud 2008), although sediments still remain a net sink for surface primary production.

Benthic oxygen uptake is often used as a robust proxy for total benthic mineralisation (Glud et al. 2016; Jahnke et al. 2000; Stahl et al. 2004b), and this method is particularly applicable in cohesive sediments (Glud 2008). The rate of oxygen uptake, or consumption, is determined by the type of sediment; the presence, quality (labile vs refractory) and quantity of organic matter in the sediment; and the organisms that live on and within the sediment (from microorganisms and meiofauna to macrofauna). Total Oxygen Uptake (TOU) measurements include the total oxygen consumption by the entire benthic community, including respiration from macrofauna, meiofauna and microorganisms (i.e. biological oxygen demand), as well as from reoxidation of reduced compounds in the sediment (i.e. chemical oxygen demand). Hence the TOU includes both diffusive and advective (e.g. bioirrigation and bioturbation) transport processes for oxygen. In contrast, Diffusive Oxygen Uptake (DOU) rates are representative of the diffusive oxygen exchange across the sediment–water interface (Fischer et al. 2009; Jorgensen and Revsbech 1985; Rabouille et al. 2003; Rasmussen and Jorgensen 1992), related to microbial respiration and chemical oxidation (Glud et al. 2016; Revsbech 1989a). The difference between TOU and DOU can be used to ascertain the relative contribution of the fauna mediated oxygen uptake (FOU), which both refers to the respiration and activity of the macrofauna itself, as well as the stimulation of microbial respiration within the sediments due to the introduction of oxygen-rich water into deeper sediment layers through bioirrigation and bioturbation (Glud 2008; Glud et al. 2016; Kristensen et al. 2012). The contribution of FOU to TOU is typically higher in coastal, slope and shelf sediments, compared to deep-sea sediments where the abundance of macrofauna is lower (Rabouille et al. 2003; Wenzhofer and Glud 2002). TOU can be measured through either ex situ whole core incubations, in situ chamber lander incubations or through non-invasive in situ Eddy Covariance lander measurements (e.g. Berelson et al. 2003; Berg et al. 2007; Rovelli et al. 2015). However, studies comparing ex situ incubation set ups with in situ measurements have found that the former can be representative of the benthic study site, provided they are run at in situ temperature and the overlying incubated water is well mixed (Lansard et al. 2003; Tengberg et al. 2004). This is typically only valid for waters shallower than 1000 m depth, as the change in pressure and temperature is less likely to affect oxygen measurements carried out on board (Glud 2008; Rabouille et al. 2003).

Advective permeable sediments are estimated to cover over 70% of the coastal shelf (de Haas et al. 2002; Glud 2008; Hall 2002), yet benthic process studies on sandy sediments are relatively sparse compared to cohesive sediments, partly due to the difficulty in sampling intact and undisturbed sand cores (Jahnke et al. 2005; Rao et al. 2007). In addition, ex situ incubation experiments designed for cohesive sediment dynamics can be less effective in permeable sediments as they may restrict advective flow and hence affect solute transport and the distribution of these compounds within the sediment (Ehrenhauss et al. 2004; Huettel and Webster 2001; Tengberg et al. 2004). In core incubations, well mixed overlying water is particularly important for permeable sediments, where advective flow determines much of the benthic biogeochemistry (Forster et al. 1996; Tengberg et al. 2005). Despite the research bias on cohesive sediments, it is vital to understand how sediment dynamics, such as oxygen consumption and carbon sequestration, change among different sediment types, particularly considering the spatial extent of permeable sediments on continental shelves. Seasonality will influence sediment reactivity, as, alongside temperature changes, variability in the flux of organic matter to the seafloor, will stimulate respiration and oxygen consumption through remineralisation and redox reactions. The presence and activity of benthic organisms, which vary with sediment type, will also directly impact carbon cycling.

**Fig. 1:**
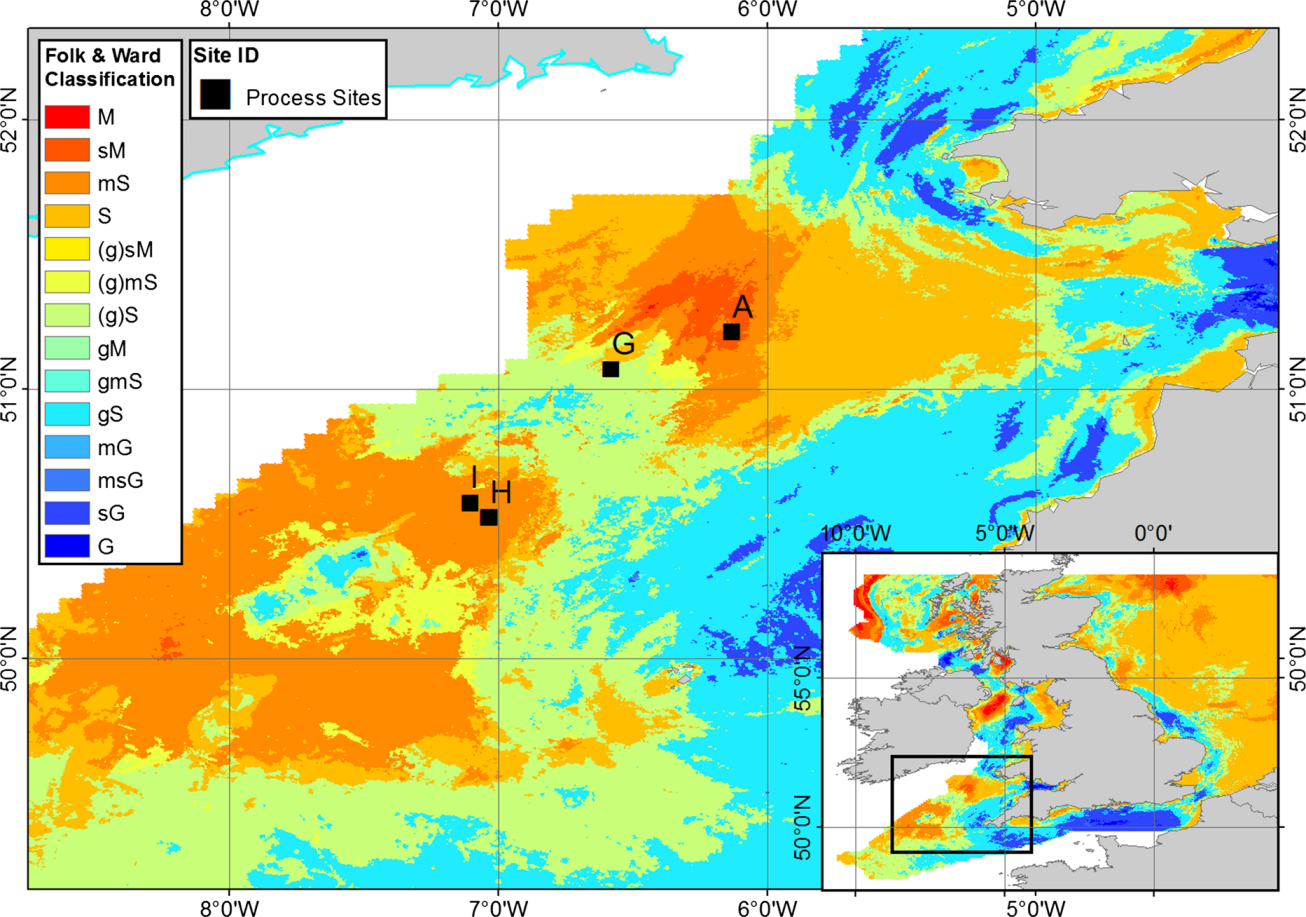
Spatial map of the Celtic Sea showing sediment type for UK shelf, and the four benthic sites in the Celtic Sea. Sediment type determined using simplified Folk textural classifications based on BGS surface maps (Folk 1954; Stephens 2015; Stephens and Diesing 2015)

Most studies that have examined the vertical distribution of oxygen and variability in uptake rates in continental sediments have focused on single study sites (Janssen et al. 2005; Stahl et al. 2004c) or sediment types (Forster et al. 1996; Huettel et al. 1996; Jahnke et al. 2005; Stahl et al. 2004c); or one sampling time point along a transect in a specific study area (Hartnett et al. 1998). Very few studies (Berelson et al. 2003; Fuchsman et al. 2015; Neubacher et al. 2012) have systematically investigated seasonal variability of benthic oxygen dynamics and carbon cycling across several sediment types. The Celtic Sea provides an accessible shelf habitat, with a variety of sediment types covering a range of shelf sediments (see Fig. 1), and is representative of benthic shelf faunal assemblages (Thompson et al. submitted, this issue). Here we capture the seasonal variability of four different continental shelf sediments in the Celtic sea (water depth ~100 m), encompassing a gradient of cohesive to permeable sediments. Three incubation techniques and two microelectrode profiling campaigns provide temporal and spatial resolution of shelf sea benthic oxygen dynamics.

## Methods

Four benthic process sites in the Celtic Sea (Fig. 1) were identified for their different sediment properties (see Table 1; for details on site selection and description see Thompson et al., this issue). The four sites represented a gradient of sediment types from muddy cohesive to sandy permeable sediment, with site A as the most cohesive (muddy), site G as the most permeable, with sites H and I being intermediates (muddy sand and sandy mud, respectively). As Site G is the most permeable site (based on porosity and grainsize), for the purpose of this study it is referred to as the ‘sandy’ site to contrast it from the other more cohesive benthic process sites. All four process sites were sampled over three seasons (March—winter/pre bloom; May—spring/bloom; August—summer/post-bloom) in 2015, where bottom water temperature did not vary (annual average 9.3–10.13 °C (Thompson et al. submitted, this issue)) with season. An additional cruise took place in March 2014 (pre-bloom).

**Table 1:** Basic sediment properties for the four benthic process sites, showing average porosity,  % sand and  % clay

Process site	Porosity	% Sand	% Clay	Central point location	Bottom water temp (°C)
Site A (mud)	0.68 ± 0.05	35.02	64.97	51°12.6754N −6°8.0277E	9.56 ± 0.22
Site G (sand)	0.44 ± 0.07	83.21	12.85	51°4.3569N −6°34.866E	9.32 ± 0.09
Site H (muddy sand)	0.55 ± 0.02	76.38	22.85	50°31.3329N 7°2.142E	10.13 ± 0.61
Site I (sandy mud)	0.58 ± 0.05	65.67	34.30	50°34.5557N −7°6.3161E	10.13 ± 0.61

Table based on data from Silburn et al. (accepted, this issue) and Thompson et al. (submitted, this issue)

Sediment was collected as described in detail in Thompson et al. (this issue) using a NIOZ corer (Haja Boxcorer (K16), 32 cm internal diameter, 0.08 m^2^ total area including overlying water) at each benthic process site. Megacore tubes (10 cm internal diameter, 60 cm height) were used to subsample from each NIOZ core for incubation measurements (experiments A and B: *n* = 6; sediment depth 30–38 cm). Smaller sub-cores (5.7 cm internal diameter, 30 cm height) were collected for experiment C (sediment depth 12–20 cm). An additional NIOZ core was collected for immediate oxygen microprofile low-resolution measurements. The six cores for incubation experiment A were re-aerated overnight post-incubation and oxygen microprofile high-resolution measurements were made in each core the next day.

### Total oxygen uptake (TOU) rates

Three different incubation experiments were used to monitor changes in the concentration of oxygen in the overlying waters. All experiments described below were run in a constant temperature (CT) room set to 10 °C, simulating the average in situ temperature of the bottom water of each sample site.

### Incubation experiment A

At each process station, megacore tubes (*n* = 6) were subsampled from six different NIOZ corer casts. Each core was immediately brought into the CT room and placed in a large incubation barrel around a central magnetic motor. Each core was fitted with a small central stirring magnet to ensure a well-mixed overlying water layer (typical height ~28 cm) and kept at the in situ temperature. Cores were sealed with airtight lids (making sure no air bubbles were trapped in the cores) and left incubating for up to 36 h in the dark. During the incubations, the oxygen concentrations in the overlying water were recorded continuously using a non-invasive fibre optic oxygen meter (Firesting, PyroScience Sensor Technology). The fibre optic sensors measured the oxygen concentration through a small transparent window in the lid, with an oxygen optode sensor spot attached to the inside which was exposed to the well mixed overlying water in the core. The oxygen optode sensor spots were calibrated in a separate container, prior to each incubation, using a two-point calibration (0 and 100% air saturation) with bottom water (ensuring correct in situ temperature and salinity). At the end of the incubation, the cores were opened and gently re-aerated prior to microprofiling. The TOU was calculated from the slope of the oxygen concentration change over time in the overlying water, using a linear approximation of the initial (10–15%) decrease in the oxygen concentration (Glud 2008; Tengberg et al. 2004).

### Incubation experiment B

As in experiment A, megacore tubes (*n* = 6) were subsampled from the NIOZ corer and brought into the CT room, where they were sealed with individual air-tight lids, stirred continuously via geared 12v motors connected to discs (8 cm diameter, 1 cm thick) (Khalili et al. 2008) revolving at 40 rpm (Glud et al. 2008), and maintained in the dark. A pilot study on advective sediments showed that increasing stirring speed to 80 rpm had no significant (*p* > 0.05) effect on the resulting nutrient efflux rates (Fig. S1). Cores were aerated through lid ports for 15 min every 3 h for 18 h, after which the cores were then resealed and incubated for 6 h. Oxygen concentrations were measured non-invasively at the start (t_0_) and hourly through to the end (t_6_) of this six-hour incubation, using calibrated fibre optic sensors connected to a Fibox 3 oxygen meter (PreSens Precision Sensing GmbH) (calibrated as above). Oxygen uptake rate was calculated from the slope of the line generated by the hourly measurements using random effects linear models (Mayor et al. 2012) to determine the best fit regression.

### Incubation Experiment C

Cores (*n* = 12; internal diameter ~5 cm) were subsampled from the NIOZ cores and immediately sealed with rubber bungs. Oxygen samples were taken from four cores at the start of the incubation prior to destructive sampling and the remaining eight cores were sampled ~30–40 h later. Magnetic bars (40 mm length × 6 mm diameter) were suspended in the overlying water of incubated cores and agitated externally by electromagnets (at 2 Hz) to induce a gentle stirring motion to replicate in situ water movement without resuspending sediment. After incubation, sediment and overlying water were homogenised and subsamples of the slurries were collected in 125 mL glass bottles, sealed with crimp-top septa and poisoned using 0.5 mL of 50% ZnCl_2_ solution. These sub-samples were stored in the dark at room-temperature until analysis by membrane inlet mass spectrometry (MIMS) (Tortell 2005). Analytical grade water (18.2 MΩ Millipore-MilliQ) and seawater (salinity 37) were equilibrated with air at 25 °C and used as standards, given temperature and salinity dependence of O_2_ solubility (Garcia and Gordon 1992). Oxygen uptake was calculated as the difference in O_2_ concentration between incubated and initial samples. Sediment homogenization would introduce a dilution of the overlying water O_2_ with subsurface, low-O_2_ porewaters. Since we were interested in O_2_ change in the overlying water, we corrected for this dilution by taking into account the porewater volume (volume of bulk sediment × porosity) and volume of overlying water (height of overlying water × cross-sectional area of core).

### Oxygen micro-profiling—oxygen penetration depth and diffusive oxygen uptake

Clark-type oxygen microelectrodes (Unisense), equipped with an internal reference and a guard cathode, were used for measuring sediment oxygen microprofiles (Revsbech 1989b) in retrieved sub-cores from the NIOZ corer. The sensors were connected to a picoampere meter (PA2000, Unisense) and the sensor signal was transferred to a PC via an analogue–digital converter. Two different types of oxygen microelectrodes were used, one with a tip diameter of 500 µm for rapid low-resolution profiling (1 mm increments) and another type with a finer tip of 50 µm for high-resolution profiling (200 µm increments). Low-resolution oxygen profiles were recorded in cores immediately after sub-sampling from the NIOZ corer to rapidly measure the oxygen penetration depth (OPD) within the sediment. Further high-resolution microprofiles (*n* = 5) were taken post-incubation (after aeration) in the experiment A cores (*n* = 6), but only in the cohesive sediments (Sites A, H, I). High-resolution profiling at site G resulted in sensor breakage, thus the larger 500 µm sensor was used to profile at 1 mm increments in the permeable sediment (Site G). OPD was determined as the first point in the tail of the profile where the oxygen concentration remained consistently low and at the same value (anoxic), according to published methods (Rabouille et al. 2003).

DOU rates were measured from the high-resolution oxygen profiles (cohesive sediments A, H and I), using Fick’s first law of diffusion (Glud 2008; Rasmussen and Jorgensen 1992), as follows:

$$DOU = D_{0 } \frac{dC}{dz}$$

where *D*_*0*_ is the oxygen molecular diffusion coefficient (based on temperature and salinity), *C* is the concentration of oxygen at position *z* in the diffusive boundary layer (DBL). The DBL was determined as the distinct linear portion in the microprofile just above the sediment–water interface. DOU was not calculated for site G or on the low-resolution profiles.

### Fauna mediated oxygen uptake

fauna mediated oxygen uptake was calculated by subtracting the DOU from the TOU. The relative contribution of DOU to TOU was calculated as DOU/TOU (Glud 2008) for all the cohesive sediment sites (A, H, I) for all three seasons.

### Macrofauna/meiofauna biomass

Macrofauna were sampled through sieving (1 mm mesh) of 5 replicate NIOZ cores at each of the four benthic sites on each of the cruises, before being counted and weighed. Sub-samples (*n* = 3) for meiofaunal analysis were collected from NIOZ cores using 50 ml syringe cores at each site for the first two cruises (March 2014; DY008 and March 2015; DY021). Further details on all biological sampling and processing can be found in Thompson et al. (submitted, this issue).

### Statistical analysis

ANOVA tests were run in the statistical programme R (R Development Core Team 2015) to determine the difference in TOU and DOU rates and OPD over season (Month/Cruise) and sediment type (Site) for oxygen incubation experiment A, due to the high number of replicates with this set up. Each ANOVA was run through Tukey’s post hoc analysis to determine the important terms and interactions, and sediment (Site) and season (Cruise) were included as an interaction in all ANOVAs. Full results of the statistical analyses can be found in the supplementary material (Tables S1–S3).

## Results

### Total oxygen uptake

**Fig. 2:**
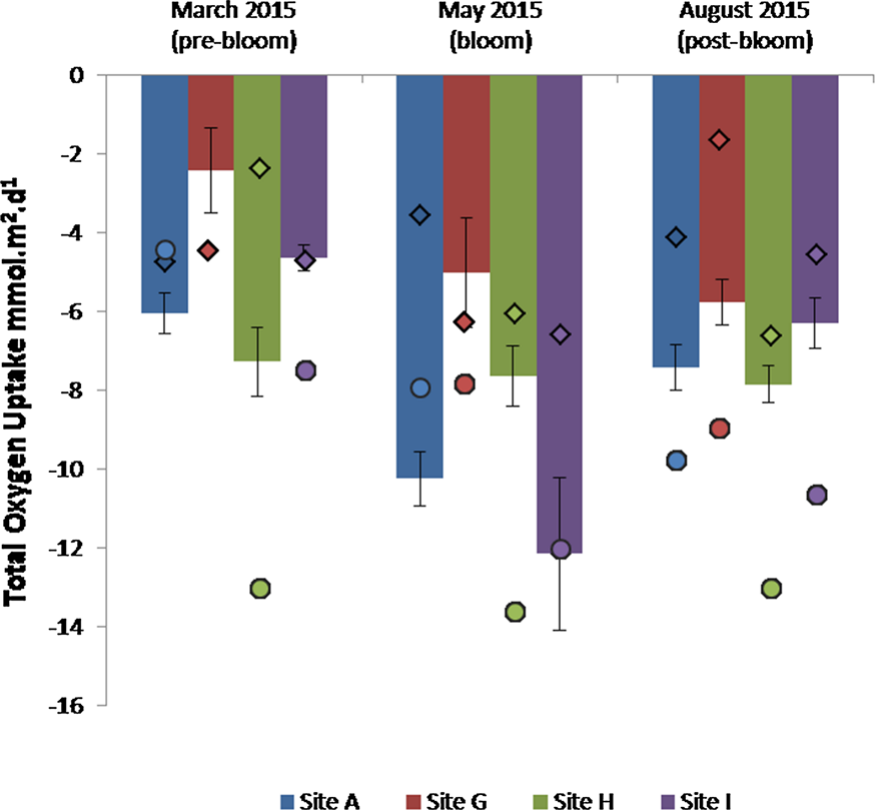
Total oxygen uptake rates taken from experiment A (*solid bars*) for each benthic site for the three 2015 cruises. *Closed circles* represent total oxygen uptake values from experiment B, *diamond symbols* represent total oxygen uptake values measured in experiment C. The *error bars* show the standard error for experiment A

TOU rates varied between sites and ranged from 2 to 14 mmol m^−2^ day^−1^. There were significant differences in oxygen uptake with sediment type (Site) across all seasons with the permeable sediment site (G) consistently (*p* < 0.001) showing the lowest oxygen uptake rates for experiment A (Fig. 2). There was a strong seasonal difference (*p* < 0.001), with oxygen consumption in the cohesive sediments (A, H, I) peaking during the May bloom cruise, with the highest measurements at −12 mmol m^−2^ day^−1^ at Site I for experiment A, and −14 mmol m^−2^ day^−1^ at Site H for experiment B. The TOU rates between sediment types were significantly different (*p* < 0.001), although there appeared to be greater variability between sites in the May bloom cruise. TOU rates in the permeable sediment (Site G) peaked in May and dropped in August (Fig. 2), however, oxygen uptake rates in experiment C were generally lower than those measured in experiments A and B. Higher TOU rates (−4 to −14 mmol m^−2^ day^−1^) were measured in experiment B (Fig. 2), particularly at site H, across all seasons, and permeable sediment Site G again had the lowest oxygen uptake rate in bloom and post-bloom conditions. Statistical differences between sites and season can be seen in Table S1.

**Fig. 3:**
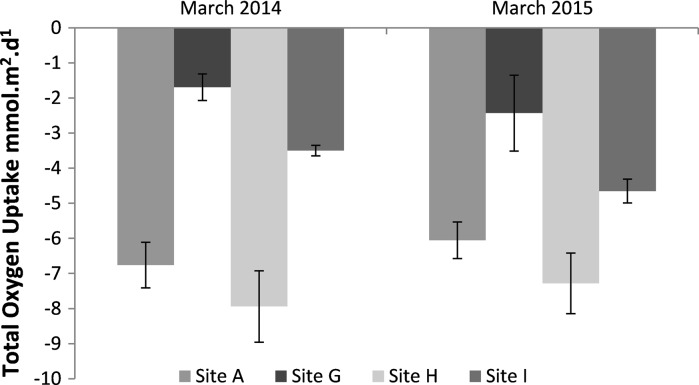
Total oxygen uptake rates taken from experiment A for the four benthic sites in March 2014 and March 2015. The *error bars* show the standard error

TOU rates measured during the two pre-bloom March cruises (2014 and 2015) showed similar trends despite being a year apart (Fig. 3), with lowest rates in the permeable sediment (G), and no significant difference between the two years (*p* > 0.05). The highest rates were recorded in the cohesive sediments at site A and site H, and the difference between the sediment types was significant (*p* < 0.001) (see Table S2).

### Diffusive oxygen uptake rates

**Fig. 4:**
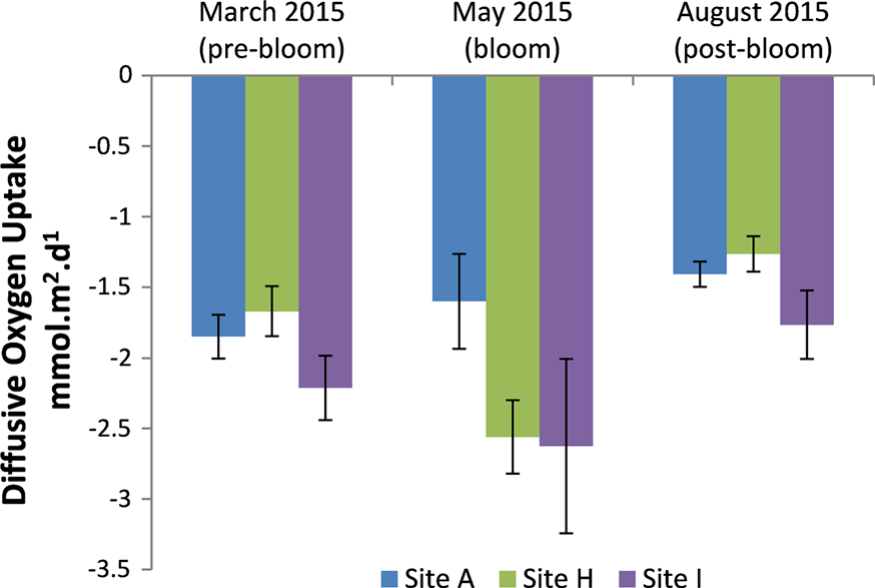
Diffusive oxygen uptake rates taken from experiment A for the cohesive benthic sites for the three 2015 cruises. The *error bars* show the standard deviation for experiment A. Diffusive oxygen uptake rates were calculated from sediment oxygen profiles using Fick’s first law of diffusion

Diffusive oxygen uptake (DOU) rates were calculated from the high-resolution oxygen microprofiles for the cohesive sediments (A, H, I). DOU rates were lower in range than TOU rates for the cohesive sites, ranging from −1.2 to −2.5 mmol m^−2^ day^−1^. DOU changed significantly with season (Fig. 4, *p* < 0.01), whereas there was no significant difference between sediment type (*p* > 0.05). The difference in DOU by season was underpinned by the significant difference (*p* < 0.05) between Site I during bloom and Site H post-bloom. There was no other significant difference in DOU rates between sites or season.

### Relative contributions to oxygen uptake

**Fig. 5:**
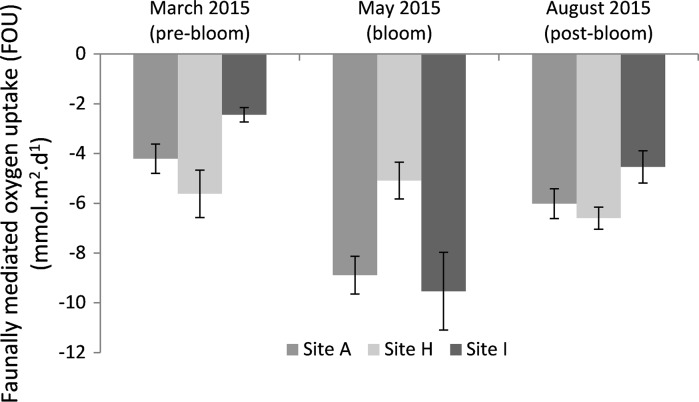
Faunal oxygen uptake (FOU; calculated as TOU-DOU) shows the relative contribution of faunal mediated respiration to total oxygen consumption across the three seasons for the cohesive sediment sites (A, H, I) over the seasons

fauna mediated oxygen uptake was calculated in the cohesive sediments (Sites A, H, I; Fig. 5) and showed a significant interaction between season and site (*p* < 0.001) (Table S2). FOU did not change between seasons at Site H (−5 to −6 mmol m^−2^ day^−1^), in contrast to Site A and Site I, which both peaked during the May bloom (Fig. 5). The lowest FOU rates for Sites A and I were seen in March (pre-bloom) (−4 mmol m^−2^ day^−1^ for Site A and −2 mmol m^−2^ day^−1^ for site I). There was no significant difference in FOU between the two pre-bloom measurements in 2014 and 2015 (*p* > 0.05), but oxygen uptake rates between sites was significantly different (*p* < 0.01), with lowest rates seen consistently at Site I across both sampling years for March (Fig. S2).

**Fig. 6:**
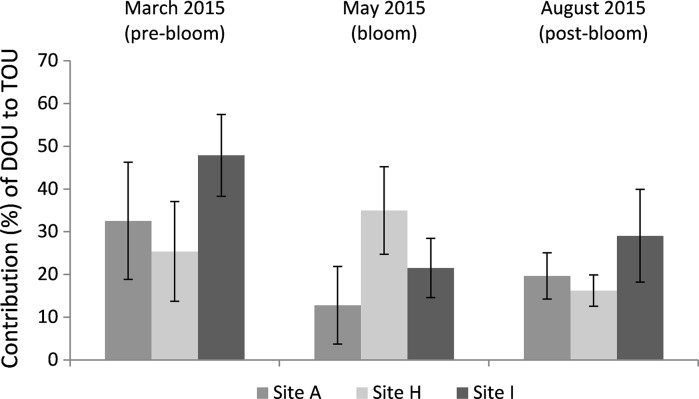
The relative contribution (%) of diffusive oxygen (calculated as DOU/TOU expressed as a percentage) changes between benthic sampling site for the cohesive sediments and with season. Methods based on Wenzhofer and Glud (2002), Glud (2008), Glud et al. (2016)

Oxygen uptake ratios (DOU/TOU) across the cohesive sediment sites (A, H, I) for the three seasons are presented in Figs. 6 and S3. Faunally mediated respiration showed a greater contribution than DOU at all sites, particularly at Site A and I during the bloom season, whilst Site H showed a slight decrease in FOU and/or increase in relative DOU during the bloom (Figs. 6, S3). The highest contribution of DOU to TOU was seen during pre-bloom sampling, particularly for Site I.

### Oxygen penetration depth

**Fig. 7:**
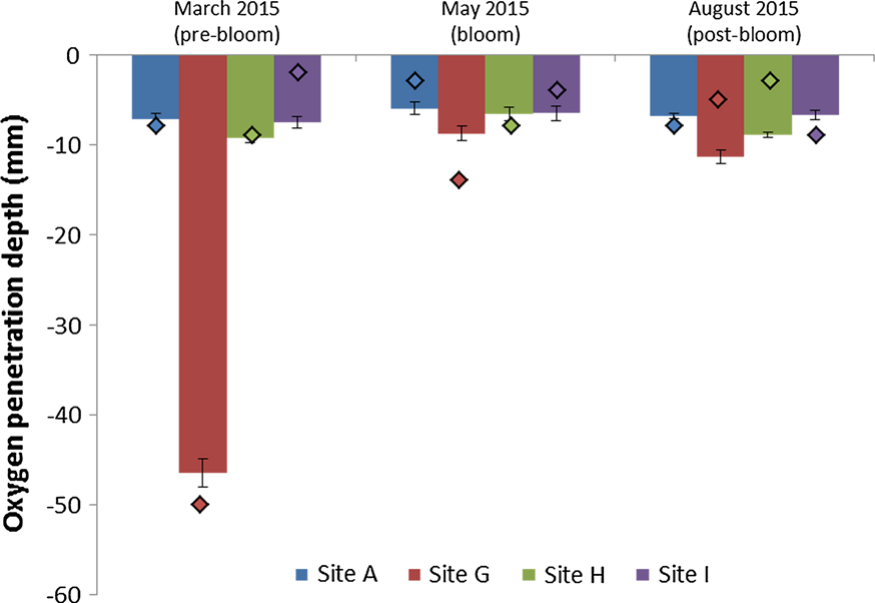
Oxygen penetration depths taken from oxygen profiles on post-incubation cores (*n* = 6, *solid bars*, ~5 profiles per core) at 200 µm increments and on fresh cores (*filled diamonds*) at 1 mm increments. The *error bars* show the standard error for incubation core experiment A

**Fig. 8:**
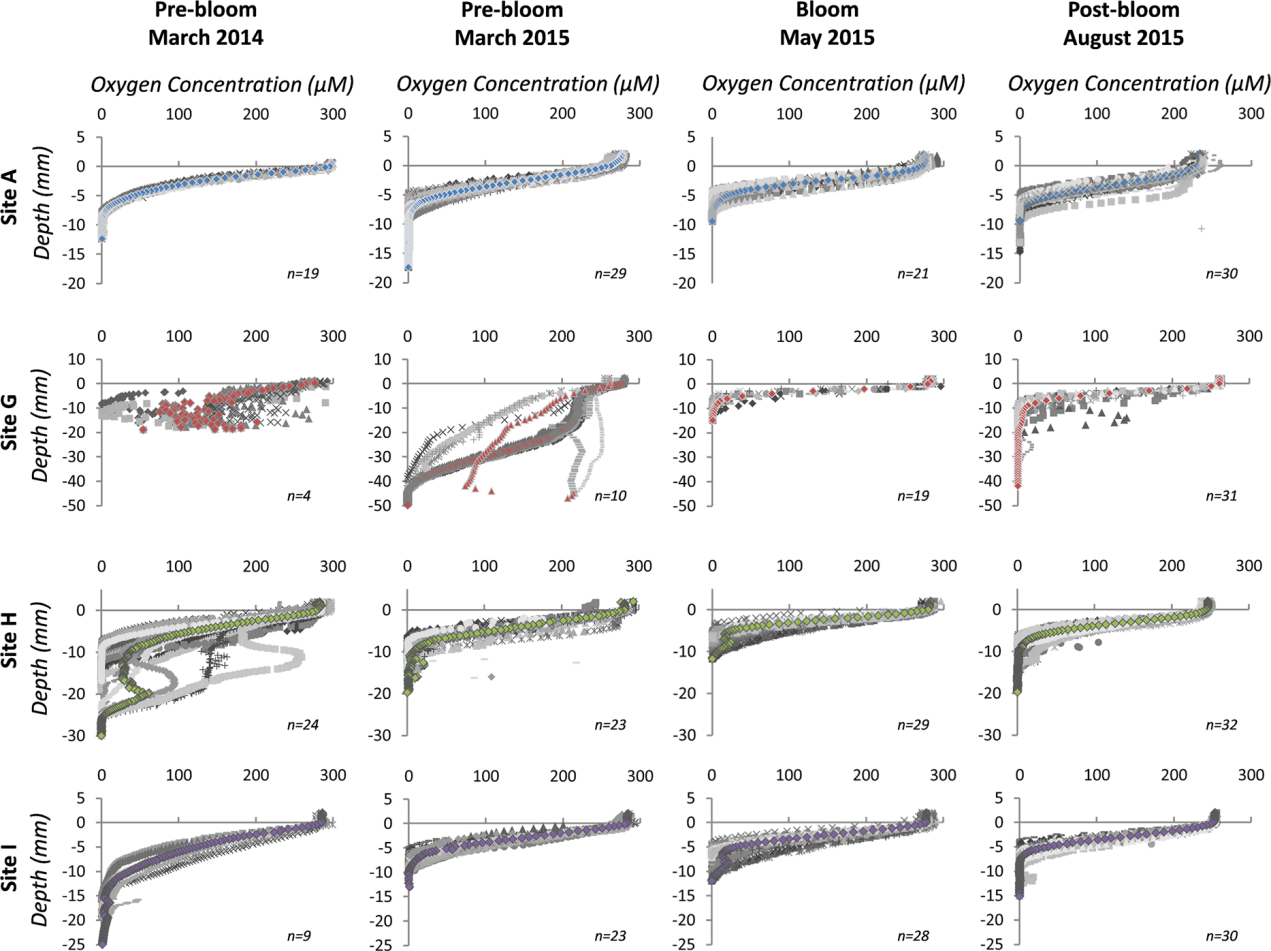
Oxygen sediment profiles for each benthic process site from the high-resolution (200 µm increment) measurements. *Filled diamonds* represent the mean for all fine profiles at each site (*blue* = site A, *red* = site G, *green* = Site H, *purple* = Site I) for each cruise. *Each graph* shows the number of profiles (*n*) per site per cruise, and the average is taken from these. (Colour figure online)

Oxygen penetration depth showed clear significant differences between sediment type (Site; *p* < 0.001) and season (*p* < 0.001), much of this is underpinned by the deep OPD (~46 mm) at permeable sediment Site G (Fig. 7) in the pre-bloom March 2015 cruise. In contrast, all three cohesive sediment sites (A, H, I) had an OPD of <1 mm. Site G consistently had the deepest OPD across all seasons, and the low-resolution and high-resolution measurements show the same overall trend (Figs. 7, 8, S4). In the low-resolution measurements, OPD was not always consistent with the high-resolution measurements (Fig. S4). Despite this, the general trend showed a shoaling of OPD across all sites during the May bloom sampling period. The high-resolution measurements show evidence of burrows in the cohesive sediment sites, particularly at Sites H and I (Figs. 8, S4). The concentration of oxygen in the overlying water differed slightly between the low-resolution and high-resolution microprofile measurements.

## Discussion

There were clear seasonal trends within each benthic process site and each site shows differing oxygen dynamics. It is clear that, despite the different experimental methodologies employed during this study, much of the oxygen dynamics are determined by the type of sediment (Silburn et al. accepted, this issue), with the most permeable sediment (Site G) showing the deepest OPD (Fig. 8), and the lowest TOU rates. In general, TOU was highest in May, coinciding with the flux of organic matter from the phytoplankton bloom recorded in late April-early May 2015, seen as a peak in chlorophyll-a at the sea surface (Kitidis et al. 2017; Thompson et al. submitted, this issue), and the associated increase in organic matter processing by the benthos. Despite the change in season, the bottom water temperature was relatively constant (Table 1; see Thompson et al., submitted, this issue), ruling out any temperature-driven changes in oxygen.

Oxygen dynamics at the studied locations were likely dominated by heterotrophic metabolism (respiration by meiofauna, macrofauna and microorganisms) and chemical oxidation, given that the water depth (~100 m) was sufficient to preclude photosynthesis at the seafloor. The FOU rates (TOU-DOU), which provide information on the relative contribution of macrofauna and meiofauna to TOU (Glud 2008; Wenzhofer and Glud 2004), suggested that there was a peak in faunal respiration and activity at Sites A and I. This was during the May bloom, likely reflecting their response to the associated arrival of organic matter. Similarly, sediment anammox and denitrification showed a dramatic increase during the May bloom at Sites H and I (Kitidis et al. 2017). Faunal measurements support these site differences, with biomass and mean abundance being greatest at Sites A and I (Table S4; for details on sampling see Thompson et al., this issue). In addition, Site A had the highest percentage of organic carbon and chlorophyll in the surface sediment, which both peaked in May (Fig. S5). The pre-bloom sampling in March 2014 and 2015 indicated that microbial respiration played a greater role than macrofaunal and meiofaunal respiration and activity at this time point (higher DOU fraction relative to TOU). This is consistent with the understanding that faunal activity increases in response to the surface water bloom. In contrast, TOU in the most permeable sediment, Site G, remained consistently low throughout our sampling programme, linked to the lowest abundance and biomass of macrofauna in all benthic sites (Table S4). Nevertheless, OPD was much shallower during May compared to March, suggesting these sediments had also undergone reactive changes in response to the bloom which were not captured in the incubation measurements, potentially because very little labile carbon was left in the sediments at the time of incubation.

Core incubation measurements are most representative of the in situ conditions if they are collected from shallow depths, as seen here with a depth of ~100 m, and the cores are kept at the representative in situ water temperature with a well-mixed overlying water layer (Glud 2008; Tengberg et al. 2004). Permeable sediments incubations have a tendency to favour diffusive processes; replicating the advective processes that dominate the biogeochemistry of permeable sediments ex situ incubation set ups remains methodologically challenging (Fuchsman et al. 2015; Glud 2008; Jahnke et al. 2005; Lohse et al. 1996; Rao et al. 2007; Viollier et al. 2003). Despite differences in the absolute values of TOU, the three incubation set-ups revealed the same general trends in space (sediment type) and time (season).

Oxygen sediment profiles, obtained from different microelectrodes, provide details on OPD, and the organic matter oxidation rate (Cai and Reimers 1995; Cai and Sayles 1996). Despite differences in values and increments, the overall trend of a shoaling of OPD in the May bloom cruise was consistent between the high-resolution and low-resolution measurements. This consistency was particularly noticeable in the March 2015 datasets for Site G. However, the high number of profiles taken using the high-resolution measurements on the incubated cores also demonstrates the spatial variability (heterogeneity) in the microprofile structure (Fig. 8), which was lost in the low-resolution measurements. Variability in sediment pore water oxygen concentration can be seen in Sites H and I, and is probably attributable to faunal burrows (Grenz et al. 2003; Rabouille et al. 2003; Viollier et al. 2003). Infaunal activity within burrows (bioturbation and bioirrigation), in addition to respiration, will directly increase the TOU rate (Viollier et al. 2003; Woulds et al. 2007), and this was apparent in the May bloom measurements, likely due to increased faunal activity in response to the input of fresh organic matter. The difference in the oxygen concentrations of the overlying water between the high and low-resolution profiles, with the exception of Site A for the March cruises (Figs. 8, S3), can be attributed to differences in measurement conditions: the immediate use of sub-cores from the NIOZ corer for the low-resolution measurements, which were processed within half an hour of core retrieval and measured in the on-deck laboratory; whilst the high-resolution measurements were conducted on cores from incubation experiment A which were re-aerated prior to microprofiling, but kept at in situ temperature. Although the faster low-resolution measurements provided immediate data on the OPD, the high-resolution measurements increased the resolution at which spatial variability could be examined, and enabled us to discern the diffusive boundary layer (DBL) and calculate DOU rates (Glud 2008; Rasmussen and Jorgensen 1992). Both microprofile techniques consistently demonstrated that the deepest OPD was found at Site G, the most permeable sediment, and a shoaling of the OPD across all sediments during the bloom. These data support the hypothesis that biological activity and seasonality significantly influence oxygen consumption, and suggest that Site G may exhibit highest remineralisation rates in late summer (Jahnke et al. 2005). Permeable sediments are able to remineralise fresh carbon quickly due to the advective flow (Forster et al. 1996). In agreement, sediment NH_4_^+^-oxidation, denitrification and anammox rates at site G peaked during the summer cruise (Kitidis et al. 2017).

The ability to measure both TOU and DOU provides data on different diagenetic processes within the sediment (Rabouille et al. 2003; Viollier et al. 2003). Previous studies have found that the ratio of TOU to DOU (Fig. 6) is usually 3:1 or 4:1 in continental slope sediments (Lindeboom et al. 1985; Wenzhofer and Glud 2002), and 1:1 in deep-sea sediments, where the faunal contribution (captured by TOU) is much smaller (Rabouille et al. 2003). TOU rates in shelf sediments tend to be higher than in deep-sea sediments, such as in the North East Atlantic (Porcupine Abyssal Plain) where TOU rates range from 0.3 to 1.85 mmol m^−2^ day^−1^ (Stahl et al. 2004c). This study shows TOU:DOU ratios vary from 2 to 7, averaging ~5, suggesting that infaunal activity may play a particularly important role in the biogeochemistry of the Celtic Sea sediments and wider UK shelf sediments (dominated by a mixture of cohesive and permeable sediments, see Fig. 1). The DOU/TOU ratio shows the relative contribution of FOU via respiration as well as faunal mediated processes, such as bioirrigation and biodiffusion. The contribution of FOU tended to be highest in the more cohesive sediments, and varied with season, peaking for Sites A and I during the bloom where macrofaunal (Site A) and meiofaunal (Site I) biomass was high. At Site H the DOU increased during the May bloom, unlike Sites A and I where the TOU increased, thereby reducing the observed DOU/TOU ratio. This suggests that microbial processes and chemical oxidation predominated at Site H, whereas the influence of FOU was greater at Sites A and I, irrespective of season. Overall, this study suggests that diffusive processes do not generally dominate the benthic oxygen demand in cohesive sediments, supporting previous findings where macrobenthic activity can dominate oxygen uptake in shelf sediments that receive a regular (in this case annual) supply of carbon (Jahnke et al. 2005). The presence of fauna, measured here by biomass, is known to influence the difference between TOU and DOU rates in coastal sediments (Rabouille et al. 2003), with species-specific effects (Kristensen et al. 2012).

DOU was not calculated for Site G, as the microprofile increments (500 µm) were likely to be greater than the thickness of the DBL (usually 200–300 µm in shallow sediments; Wenzhofer and Glud 2004), which means the contribution of faunal mediated respiration vs DOU was not estimated. However, a previous study suggested that the importance of FOU decreases as OPD increases (Wenzhofer and Glud 2002). As Site G had the deepest OPD of all benthic sites, it is possible that the FOU contribution was much lower than in the cohesive sediments. In addition, permeable sediments have enhanced oxygenation through advective flow, with subsequently low rates of diffusive uptake through the DBL (Janssen et al. 2005).

Variations in sediment type must be taken into account when estimating benthic processes in shelf sea sediments, and this is illustrated by the results of this study. Despite the similarity in sediment type for sites H and I, clear differences in what drives oxygen dynamics were apparent, with suspected high microbial activity at site H (muddy sand, slightly more permeable sediment) and a highest faunal contribution to oxygen dynamics for site I (sandy mud, slightly more cohesive sediment). This study covers a range of sediment types found in shelf sea sediments and illustrates how relatively small differences in sediment type (such as Site H (muddy sand) and Site I (sandy mud)) can influence the oxygen dynamics. The more permeable site (H) showed less influence by faunal activity, suggesting microbial activity plays a larger role in biogeochemical processes. In contrast, the more cohesive site (I) had high meiofaunal biomass and macrofaunal abundance, resulting in higher FOU rates, suggesting faunal activity was a strong determinant of benthic oxygen demand. This has implications for understanding benthic biogeochemistry on a UK shelf sediment scale, particularly as much of the shelf sediment is made up of a mixture of permeable and cohesive sediment e.g. the fluvial sands and fine-grained sediments dominating the North Sea (de Haas et al. 2002).

Benthic oxygen uptake provides a good proxy for benthic mineralisation (Glud et al. 2016; Jahnke et al. 2000; Woulds et al. 2007), however spatial and temporal variability of oxygen dynamics may present a bias in interpretation if careful consideration is not given to the methodology used and caveats of each approach, e.g. incubations favour diffusive processes so may lead to over or underestimation in permeable sediments (Lohse et al. 1996; Tengberg et al. 2004). This study illustrates high spatial and temporal resolution of oxygen dynamics in shelf sediments, and demonstrates that general trends for seasonality (temporal variability) and different sediment types (spatial variability) can still be identified irrespective of technique. Diffusive processes dominated pre-bloom oxygen uptake, but the arrival of organic matter during the phytoplankton bloom (Kitidis et al. 2017; Thompson et al. submitted, this issue) increased the FOU in the cohesive sediments. The use of a range of different approaches, measuring TOU as an integrative measure of FOU and DOU, and DOU separately, can provide a detailed insight into oxygen dynamics between different sediments. This allows the relative importance of these two main processes to be compared across a range of sediment types and seasons. The sediment types present in the Celtic Sea are broadly representative of the wider western European continental shelf and thus the presented benthic oxygen data provides a useful insight into carbon cycling of shelf sea sediment dynamics across a range of spatial and temporal scales, with an emphasis on understanding the variations in sediment type.

## Electronic supplementary material

Below is the link to the electronic supplementary material.

Supplementary material 1 (DOCX 2275 kb)
