# Supplementary material for: Oxygen dynamics in shelf seas sediments incorporating seasonal variability
Source: Biogeochemistry. 2017 Mar 29;135(1):35–47. doi: 10.1007/s10533-017-0326-9 (PMC6961513; doi:10.1007/s10533-017-0326-9)
Supplement: Supplementary file 1 — Supplementary material 1 (DOCX 2275 kb) [file 10533_2017_326_MOESM1_ESM.docx]

**Supplementary material**

**Oxygen dynamics in shelf seas sediments incorporating seasonal variability**

Hicks N.^*1^, Ubbara, G.R.^1+^, Silburn, B.^2^, Smith, H.E.K.^3^, Kröger, S.^2^, Parker, E.R.^2^, Sivyer, D.^2^, Kitidis, V.^4^, Hatton, A^1^, Mayor, D.J.^3^ and Stahl H.^1,5^

*^1^ Scottish Association for Marine Science, Scottish Marine Institute, Oban, Argyll, PA37 1QA, United Kingdom*

*^2^ Centre for Environment, Fisheries and Aquaculture Science, Lowestoft, NR33 0HT, United Kingdom*

*^3^ Ocean Biogeochemistry and Ecosystems, National Oceanography Centre, Southampton, SO14 3ZH, United Kingdom*

*^4^ Plymouth Marine Laboratory, Prospect Place, The Hoe, Plymouth, PL1 3DH, United Kingdom*

*^5^ College of Sustainability Sciences and Humanities, Zayed University, Dubai, United Arab Emirates*

^+^ *present address: Department of Chemistry, University of Glasgow, University Avenue, Joseph Black Building, G12 8QQ*

* Corresponding author: [natalie.hicks@sams.ac.uk](mailto:natalie.hicks@sams.ac.uk)


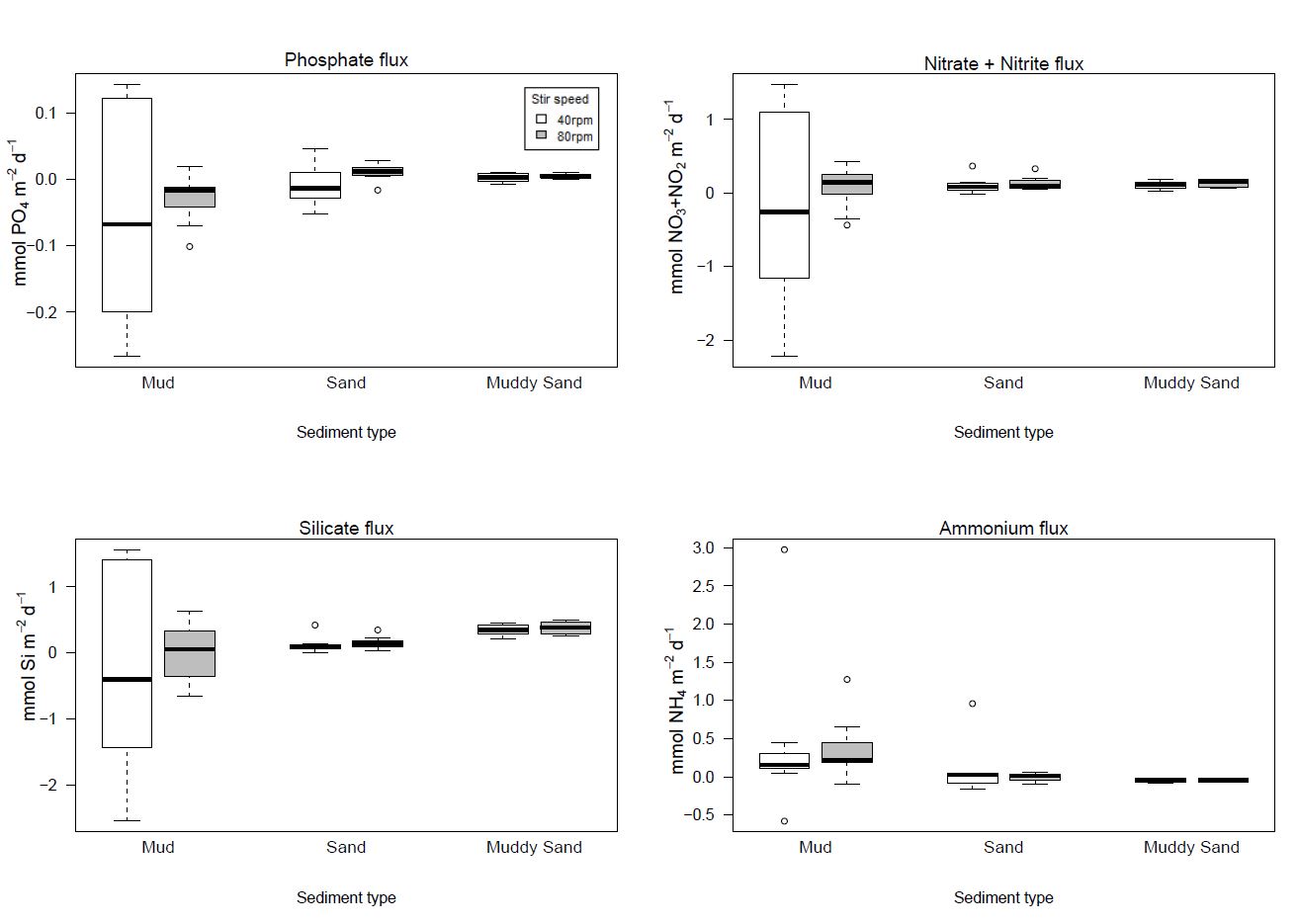


**Figure S1** Nutrient flux from sediment under different stirring speeds (40rpm, white boxes; 80 rpm, grey boxes) across three sediment types. Phosphate (top left); nitrate and nitrite (top right), silicate (bottom left) and ammonium (bottom right) fluxes showed no significant change in nutrient flux with increased stirring speed (p>0.05, unpublished data, Daniel Mayor). In this manuscript a stirring speed of 40rpm was used in Experiment B.

**Table S1a** ANOVA summary for total oxygen uptake rates (TOU~Month*Site)

|  | Df | Sum Sq | Mean Sq | F value | Pr(>F) |
| --- | --- | --- | --- | --- | --- |
| Month | 2 | 144.18 | 72.089 | 14.0815 | **1.035e-05 ***** |
| Site | 3 | 142.81 | 47.605 | 9.2989 | **4.106e-05 ***** |
| Month:Site | 6 | 119.65 | 19.942 | 3.8953 | **0.00246 **** |

**Table S1b** ANOVA summary for individual terms for total oxygen uptake rates (TOU~Month*Site)

|  |  | ***March*** | | | | ***May*** | | | | ***August*** | | | |
| --- | --- | --- | --- | --- | --- | --- | --- | --- | --- | --- | --- | --- | --- |
|  |  | **A** | **G** | **H** | **I** | **A** | **G** | **H** | **I** | **A** | **G** | **H** | **I** |
| ***March*** | **A** | *-* | *ns* | *ns* | *0.004* | *ns* | *ns* | *ns* | *0.001* | *ns* | *ns* | *ns* | *ns* |
|  | **G** | *ns* | *-* | *0.034* | *ns* | *3E-05* | *ns* | *0.016* | *1E-08* | *0.026* | *ns* | *0.01* | *ns* |
|  | **H** | *ns* | *0.034* | *-* | *ns* | *ns* | *ns* | *ns* | *0.02* | *ns* | *ns* | *ns* | *ns* |
|  | **I** | *0.004* | *ns* | *ns* | *-* | *0.004* | *ns* | *ns* | *2E-05* | *ns* | *ns* | *ns* |  |
| ***May*** | **A** | *ns* | *3E-05* | *ns* | *0.004* | *-* | *0.01* | *ns* | *ns* | *ns* | *0.047* | *ns* | *ns* |
|  | **G** | *ns* | *ns* | *ns* | *ns* | *0.01* | *-* | *ns* | *6E-05* | *ns* | *ns* | *ns* | *5E-04* |
|  | **H** | *ns* | *0.016* | *ns* | *ns* | *ns* | *ns* | *-* | *0.043* | *ns* | *ns* | *ns* | *ns* |
|  | **I** | *0.001* | *1E-08* | *0.02* | *2E-05* | *ns* | *6E-05* | *0.043* | *-* | *0.027* | *5E-04* | *ns* | *0.002* |
| ***August*** | **A** | *ns* | *0.026* | *ns* | *ns* | *ns* | *ns* | *ns* | *0.027* | *-* | *ns* | *ns* | *ns* |
|  | **G** | *ns* | *ns* | *ns* | *ns* | *0.047* | *ns* | *ns* | *5E-04* | *ns* | *-* |  | *ns* |
|  | **H** | *ns* | *0.01* | *ns* | *ns* | *ns* | *ns* | *ns* | *ns* | *ns* |  | *-* | *ns* |
|  | **I** | *ns* | *ns* | *ns* | *ns* | *ns* | *ns* | *ns* | *0.002* | *ns* | *ns* | *ns* | *-* |

**Table S2a** ANOVA summary for DOU/TOU rates (DOU/TOU~Month*Site)

|  | Df | Sum Sq | Mean Sq | F value | Pr(>F) |
| --- | --- | --- | --- | --- | --- |
| Month | 2 | 119.02 | 59.51 | 15.339 | **8.82e-06 ***** |
| Site | 2 | 6.28 | 3.14 | 0.809 | 0.451785 |
| Month:Site | 4 | 105.68 | 26.42 | 6.810 | **0.000234 ***** |

**Table S2b** ANOVA summary for individual terms for DOU/TOU ratio (DOU/TOU~Month*Site)

|  |  |  | | | | |  | | | | | | |  | | | | |
| --- | --- | --- | --- | --- | --- | --- | --- | --- | --- | --- | --- | --- | --- | --- | --- | --- | --- | --- |
|  |  |  | ***March*** |  |  | ***May*** | | |  |  | ***August*** | |  | |  | |  |  |
|  |  | **A** | **H** | **I** | **A** | **H** | | **I** | | **A** | **H** | **I** | | | |  |  |  |
| ***March*** | **A** | - | ns | ns | 0.008 | ns | | < 0.001 | | ns | ns | ns | | | |  |  |  |
|  | **H** | ns | - | ns | ns | ns | | 0.031 | | ns | ns | ns | | | |  |  |  |
|  | **I** | ns | ns | - | ns | ns | | < 0.001 | | ns | 0.018 | ns | | | |  |  |  |
| ***May*** | **A** | 0.008 | ns | ns | - | ns | | ns | | ns | ns | ns | | | |  |  |  |
|  | **H** | ns | ns | ns | ns | - | | 0.008 | | ns | ns | ns | | | |  |  |  |
|  | **I** | < 0.001 | 0.031 | < 0.001 | ns | 0.008 | | - | | ns | ns | 0.002 | | | |  |  |  |
| ***August*** | **A** | ns | ns | ns | ns | ns | | ns | | - | ns | ns | | | |  |  |  |
|  | **H** | ns | ns | 0.018 | ns | ns | | ns | | ns | - | ns | | | |  |  |  |
|  | **I** | ns | ns | ns | ns | ns | | 0.002 | | ns | ns | - | | | |  |  |  |

**Table S3a** ANOVA summary for Oxygen Penetration Depth (OPD~Month*Site)

|  | Df | Sum Sq | Mean Sq | F value | Pr(>F) |
| --- | --- | --- | --- | --- | --- |
| Month | 2 | 1138.1 | 569.06 | 172.81 | **< 2.2e-16 ***** |
| Site | 3 | 2430.0 | 810.00 | 245.98 | **< 2.2e-16 ***** |
| Month: Site | 6 | 3279.1 | 546.52 | 165.97 | **< 2.2e-16 ***** |

**Table S3b** ANOVA summary for individual terms for Oxygen Penetration Depth (OPD~Month*Site)

|  |  | ***March*** | | | | ***May*** | | | | | ***August*** | | | |
| --- | --- | --- | --- | --- | --- | --- | --- | --- | --- | --- | --- | --- | --- | --- |
|  |  | **A** | **G** | **H** | **I** | **A** | **G** | **H** | **I** | | **A** | **G** | **H** | **I** |
| ***March*** | **A** | - | 1E-07 | ns | ns | ns | ns | ns | ns | | ns | 0.0105 | ns | ns |
|  | **G** | 1E-07 | - | 1E-07 | 1E-07 | 1E-07 | 1E-07 | 1E-07 | | 1E-07 | 1E-07 | 1E-07 | 1E-07 | 1E-07 |
|  | **H** | ns | 1E-07 | - | ns | ns | ns | ns | ns | | ns | ns | ns | ns |
|  | **I** | ns | 1E-07 | ns | - | ns | ns | ns | ns | | ns | 0.0291 | ns | ns |
| ***May*** | **A** | ns | 1E-07 | ns | ns | - | ns | ns | ns | | ns | 0.0005 | ns | ns |
|  | **G** | ns | 1E-07 | ns | ns | ns | - | ns | ns | | ns | ns | ns | ns |
|  | **H** | ns | 1E-07 | ns | ns | ns | ns | - | ns | | ns | 0.001 | ns | ns |
|  | **I** | ns | 1E-07 | ns | ns | ns | ns | ns | - | | ns | 0.0012 | ns | ns |
| ***August*** | **A** | ns | 1E-07 | ns | ns | ns | ns | ns | ns | | - | 0.0036 | ns | ns |
|  | **G** | 0.0105 | 1E-07 | ns | 0.0291 | 0.0005 | ns | 0.001 | 0.0012 | | 0.0036 | - | ns | 0.002 |
|  | **H** | ns | 1E-07 | ns | ns | ns | ns | ns | ns | | ns | n | - | ns |
|  | **I** | ns | 1E-07 | ns | ns | ns | ns | ns | ns | | ns | 0.002 | ns | - |


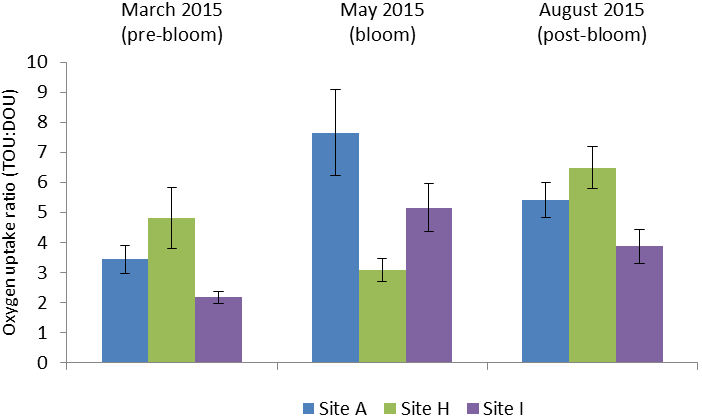


**Figure S3:** Ratio of (a) TOU:DOU indicates changes in faunal mediated respiration vs diffusive oxygen uptake across the three cohesive sediment sites with season. The relative contribution of diffusive oxygen (b), calculated as DOU/TOU, changes between benthic sampling site and with season. Methods based on (Glud 2008; Glud et al. 2016; Wenzhofer and Glud 2002).

relative contribution of macrofaunal respiration to total oxygen consumption across the three seasons for the cohesive sediment sites (A, H, I). over the seasons (a) and between annual sampling during March (b). DOU rates were not calculated for the permeable sediment site (site G).

**Figure S2** TOU-DOU shows the relative contribution of faunal mediated respiration to total oxygen consumption across the three seasons for the cohesive sediment sites (A, H, I) over the seasons (a) and between annual sampling during March (b). DOU rates were not calculated for the permeable sediment site (site G).


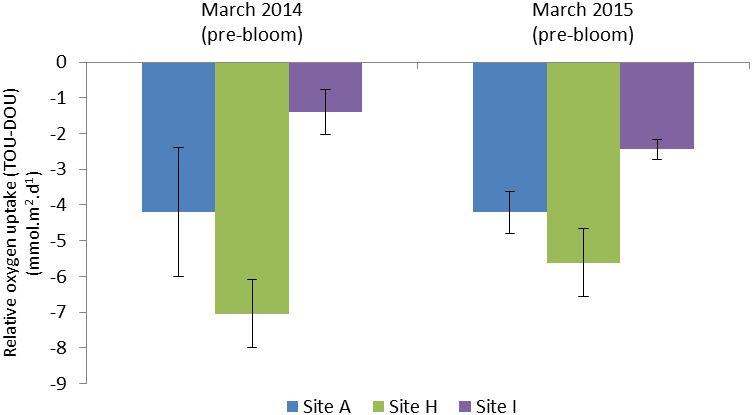


**Table S4** The macrofauna, meiofauna and microbial measurements for all four benthic sites (for details on analysis and sampling see Thompson et al, this issue). Note this table shows overall abundance and biomass measurements (with standard deviation), not seasonal variability. Macrofaunal measurements are from all four cruises, whilst meiofaunal measurements are from the two pre-bloom March cruises (DY008 and DY021).

| *Process Site* | Macrofauna (>1mm) | | | | Meiofauna (>63µm) | | |
| --- | --- | --- | --- | --- | --- | --- | --- |
|  | *Abundance (ind m^2^)* | *Wet weight biomass (g m^2^)* | | *Diversity (species)* | *Abundance (k = 1000 x ind m^2^)* | *Wet weight biomass (g m^2^)* | *Diversity (phyla)* |
| Site A  *(mud)* | 951 ± 603 | 35.7 ± 82.7 | 21.2 ± 4.8 | | 806k ± 281k | 1.13 ± 0.35 | 5.7 ± 1.3 |
| Site G  *(sand)* | 483 ± 291 | 16.0 ± 23.0 | 21.1 ± 9.1 | | 560k ± 242k | 0.68 ± 0.17 | 5.9 ± 2.0 |
| Site H  *(muddy sand)* | 1130 ± 521 | 14.0 ± 1.4 | 37.6 ± 8.1 | | 596k ± 222k | 0.73 ± 0.39 | 4.8 ± 1.2 |
| Site I  *(sandy mud)* | 1190 ± 816 | 10.2 ± 21.4 | 31.2 ± 10.6 | | 556k ± 242k | 1.14 ± 0.48 | 6.4 ± 2.0 |

**Figure S4** Oxygen sediment profiles for each benthic process site. Filled diamonds represent averages from high-resolution (200µm increment) measurements (n=12-30), open triangles represent low-resolution (1mm increment) measurements (n=1). Each row represents one site (blue = site A, red = site G, green = Site H, purple = Site I). Oxygen penetration depth was significantly different between sediment type and season.


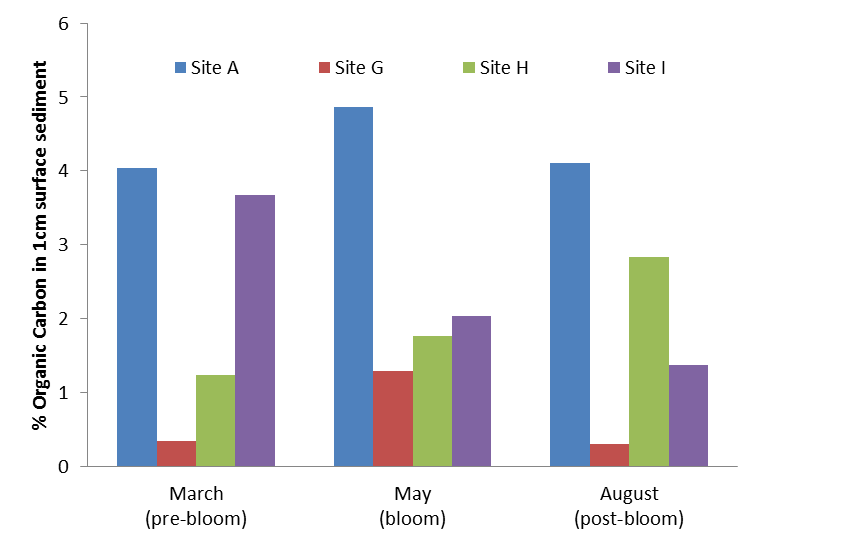

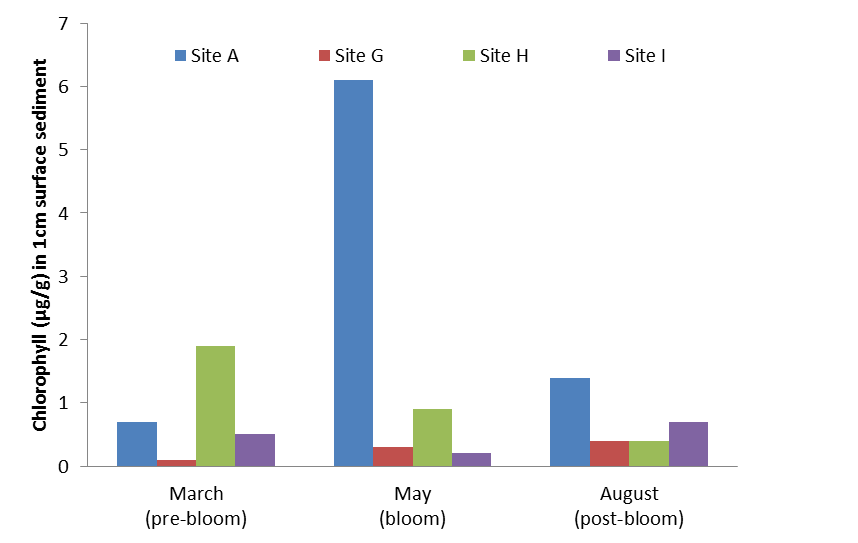


**Figure S5** Organic carbon (%) (top) and chlorophyll measurements (bottom) in surface sediment (1cm) shows seasonal changes from the four benthic sites. Site A has the largest increase in both % organic carbon and chlorophyll in May, with Site G showing an increase in % organic carbon in May. Site H shows an increase in % organic carbon with each season but a subsequent decrease in chlorophyll. Site I shows a decrease in organic carbon with each season. (% organic carbon data: Hicks, unpublished; chlorophyll: (Silburn et al. 2017))

**References**

Glud RN (2008) Oxygen dynamics of marine sediments Marine Biology Research 4:243-289 doi:10.1080/17451000801888726

Glud RN, Berg P, Stahl H, Hume A, Larsen M, Eyre BD, Cook PLM (2016) Benthic Carbon Mineralization and Nutrient Turnover in a Scottish Sea Loch: An Integrative In Situ Study Aquatic Geochemistry:1-25 doi:10.1007/s10498-016-9300-8

Silburn BE et al. (2017) Shelf Sea Biogeochemistry sediment characterisation doi:10.5285/47110529-757c-40b5-e053-6c86abc0eddc

Wenzhofer F, Glud RN (2002) Benthic carbon mineralization in the Atlantic: a synthesis based on in situ data from the last decade Deep-Sea Res Pt I 49:1255-1279
